# Emerging Tick-Borne Viruses in the Twenty-First Century

**DOI:** 10.3389/fcimb.2017.00298

**Published:** 2017-07-11

**Authors:** Karen L. Mansfield, Lv Jizhou, L. Paul Phipps, Nicholas Johnson

**Affiliations:** ^1^Animal and Plant Health Agency Addlestone, United Kingdom; ^2^Institute of Infection and Global Health, University of Liverpool Liverpool, United Kingdom; ^3^Chinese Academy of Inspection and Quarantine Beijing, China; ^4^Faculty of Health and Medicine, University of Surrey Guildford, United Kingdom

**Keywords:** tick, virus, emerging, transmission

## Abstract

Ticks, as a group, are second only to mosquitoes as vectors of pathogens to humans and are the primary vector for pathogens of livestock, companion animals, and wildlife. The role of ticks in the transmission of viruses has been known for over 100 years and yet new pathogenic viruses are still being detected and known viruses are continually spreading to new geographic locations. Partly as a result of their novelty, tick-virus interactions are at an early stage in understanding. For some viruses, even the principal tick-vector is not known. It is likely that tick-borne viruses will continue to emerge and challenge public and veterinary health long into the twenty-first century. However, studies focusing on tick saliva, a critical component of tick feeding, virus transmission, and a target for control of ticks and tick-borne diseases, point toward solutions to emerging viruses. The aim of this review is to describe some currently emerging tick-borne diseases, their causative viruses, and to discuss research on virus-tick interactions. Through focus on this area, future protein targets for intervention and vaccine development may be identified.

## Introduction

Many arthropods, including ticks, transmit diseases that cause morbidity, and mortality amongst humans, livestock, companion animals, and/or wildlife. This in turn can cause major economic costs particularly to the owners of livestock affected by disease. The relationship between the tick, its host and pathogens has been shown to be complex and each may benefit or suffer detrimental effects due to the combination of physiological and immune mediated processes each elicits during infestation and infection (de la Fuente et al., 2016). Viruses form a major constituency of the pathogens transmitted by ticks (for review see Labuda and Nuttall, 2004). Although, the presence of microorganisms in ticks appears to have little impact on the tick, presumably there is an energetic cost to harboring them and studies have demonstrated that ticks do respond in a coordinated fashion to infection with bacterial (Mercado-Curiel et al., 2011; Ayllón et al., 2015) and protozoal (Antunes et al., 2012) pathogens. Recent studies using *in vitro* models are beginning to identify transcriptional responses in ticks cell during infection with viruses (Mansfield et al., 2017).

**Figure 1:**
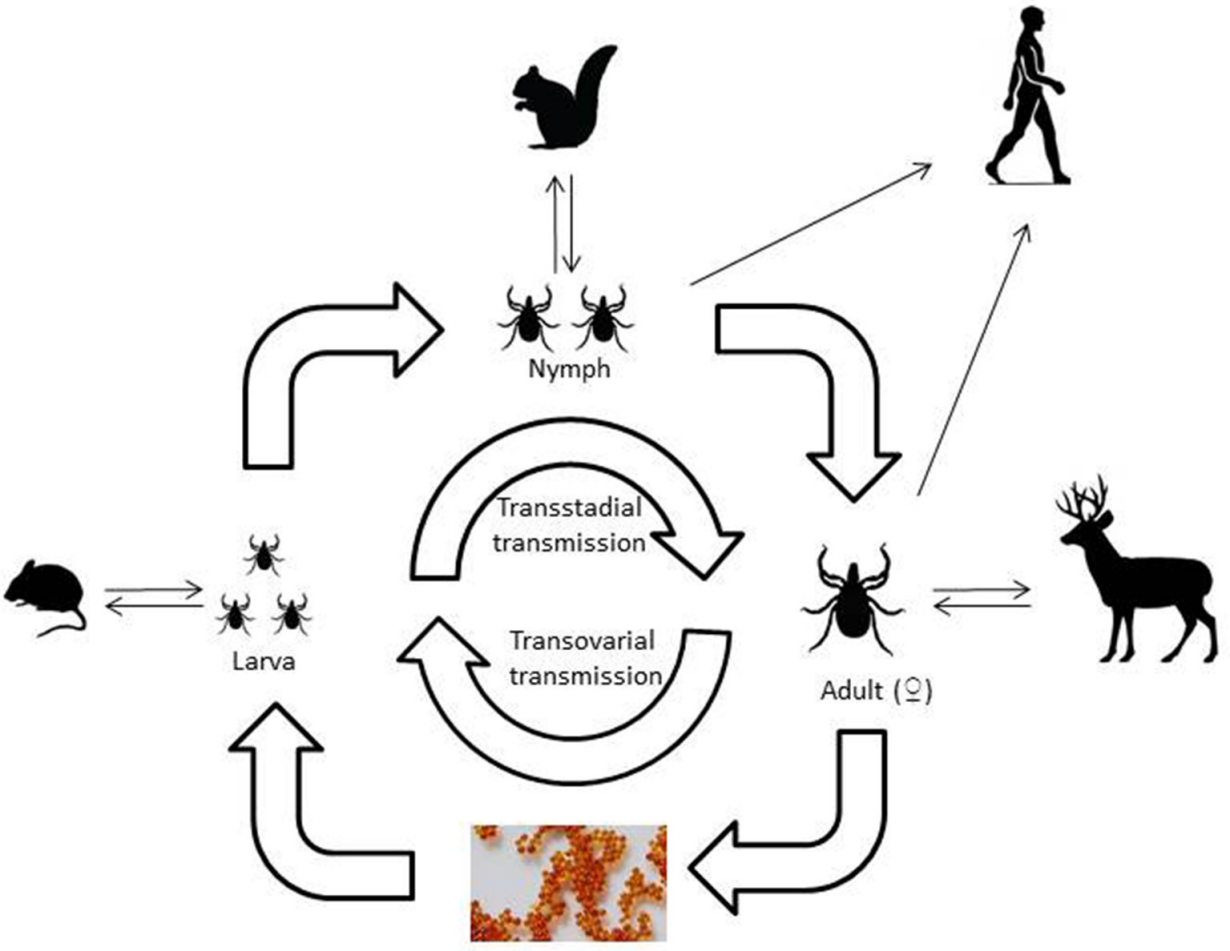
A schematic of the three host *Ixodid* tick lifecycle illustrating the type of vertebrate hosts involved in each life stage of the tick.

Viruses that are transmitted by ticks belong to a range of virus families with different characteristics and tick vectors. One of the first tick-borne viruses identified was the flavivirus, louping ill virus, the causative agent of encephalitis in sheep and grouse, a disease recognized in livestock for hundreds of years. The demonstration that ticks were the source of infection for the virus was described almost 100 years ago (Stockman, 1918). Since this time, many more pathogenic viruses have been identified around the world. However, new examples of emerging tick-borne viral diseases that affect man are constantly being reported. Another striking feature that favors pathogen transmission by ticks is the multi-stage lifecycle of ticks. Most species of *Ixodid* ticks have three states, larva, nymph and adult that each requires a blood-meal, sometimes requiring days to complete, in order to develop to the next stage. *Argasid* ticks, such as *Ornithodoros* sp. by contrast only require minutes to feed but each stage may feed multiple times. This ensures that the tick can become infected at any stage of its lifecycle and that infection persists through each developmental stage via transstadial transmission (Karbowiak et al., 2016) and then has the opportunity to transmit that virus back to a mammalian host (Figure 1). When blood feeding requires attachment to the host for a number of days, this provides ample opportunity for transfer of virus from tick to host within infected saliva. Horizontal transmission occurs following consumption of blood from an infected mammalian host, whilst co-feeding is a form of transmission that occurs when multiple ticks feed in close proximity enabling virus to transfer between ticks without infection in the host (Jones et al., 1987). Vertical transmission of pathogens between generations of ticks has been observed (transovarial transmission) for viruses such as tick-borne encephalitis virus (Reháček, 1962) and African swine fever virus (Rennie et al., 2001).

**Figure 2:**
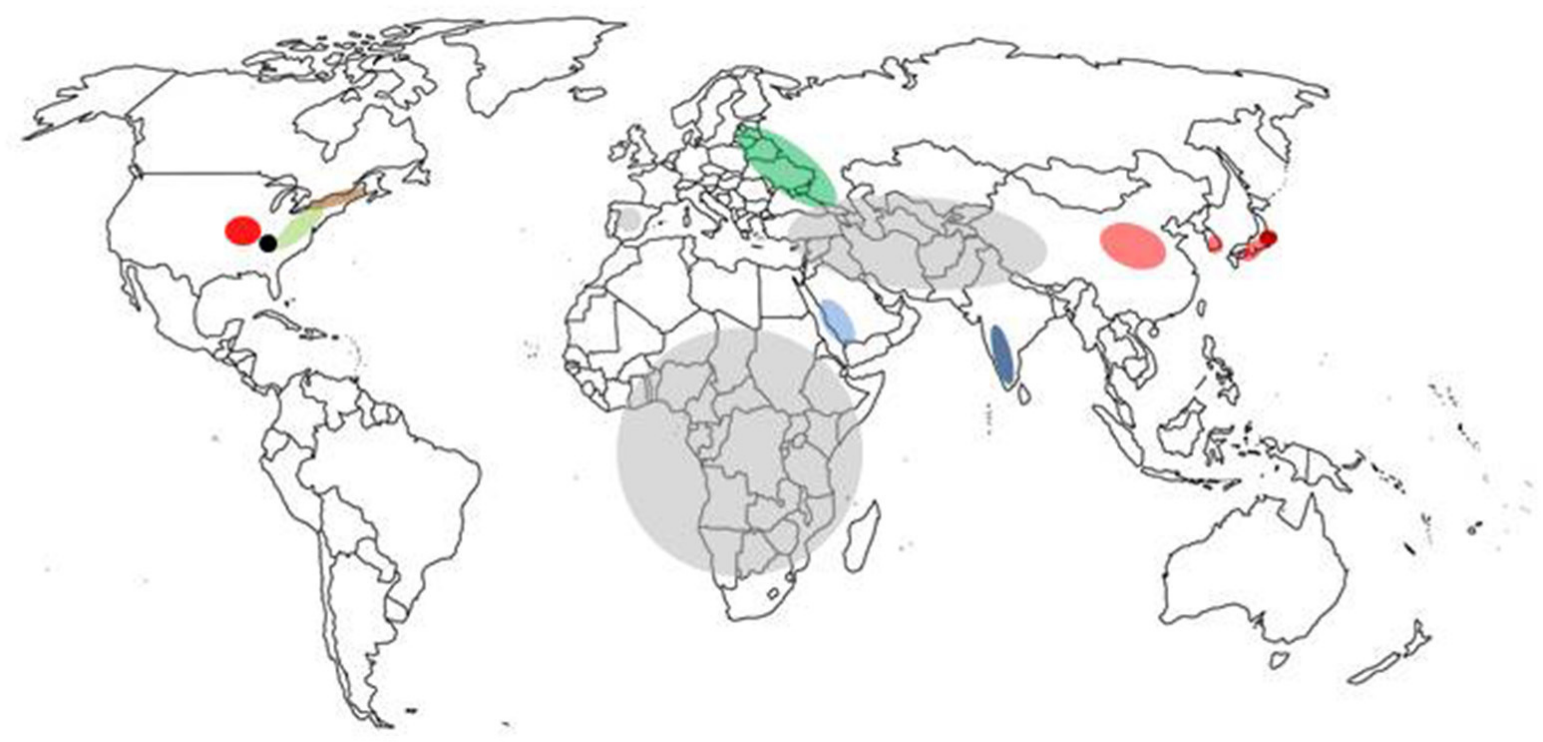
Map of the world showing location of tick-borne disease outbreaks caused by emerging viruses. The regions marked are severe fever and thrombocytopenia syndrome virus (pale red), Heartlands virus (red), Crimean-Congo haemorrhagic fever virus (gray), Kysanur Forest disease virus (blue), Alkurma haemorrhagic fever virus (light blue), Powassan virus (brown), deer tick virus (pale green), Bourbon virus (black), Tofla virus (brown), and African swine fever virus (green).

Currently, there are no effective therapeutic agents or vaccines for many tick-borne viruses with the exception of vaccines for louping ill virus, tick-borne encephalitis virus, and Kyasanur Forest disease virus, thus avoidance or control of the vector are the principal means of preventing disease. Emerging tick-borne diseases that affect humans and livestock will continue to challenge public and veterinary health. Most current research is limited to a small number of *Ixodid* tick species, mainly *Ixodes scapularis* and *Ixodes ricinus*, vectors of pathogens such as *Borrelia burgdorferi (sensu stricto* and *sensu lato), Anaplasma phagocytophilum*, and tick-borne encephalitis virus, a virus that is currently increasing in range across Europe (Mansfield et al., 2009). However, with over 900 species of ticks in the world (Horak et al., 2002), many capable of transmitting viruses, future research on the interactions between the pathogen, its vector and the mammalian host will need to consider a greater number of virus-tick associations. This article provides an overview of some of the emerging tick-borne viruses (summarized in Table 1 and located in Figure 2) that have arisen in the twenty-first century.

**Table 1:** Summary of emerging viruses discussed in this review.

**Virus**	**Abbreviation**	**Classification (family, genus)**	**Description of genome**	**Distribution**	**Primary tick vector**
Severe Fever with Thrombocytopenia syndrome virus	SFTSV	Bunyaviridae, *Phlebovirus*	Tri-segmented negative strand RNA	East Asia, North America	*Haemaphysalis longicornis*^a^
Heartland virus	HRTV	Bunyaviridae, *Phlebovirus*	Tri-segmented negative strand RNA	North America	*Amblyomma americanum*
Crimean congo haemorrhagic fever virus	CCHFV	Bunyaviridae, *Nairovirus*	Tri-segmented negative strand RNA	Africa, Central Asia, Mediterranean Basin	*Hyalomma marginatum*
Powassan virus	POWV	Flaviviridae, *Flavivirus*	Positive strand RNA	North America, Russian Federation	*Ixodes scapularis, Ixodes cookei*^b^
Deer tick virus	DTV	Flaviviridae, *Flavivirus*	Positive strand RNA	North America	*Ixodes scapularis*
Kyasunar forest disease virus	KFDV	Flaviviridae, *Flavivirus*	Positive strand RNA	India	*Haemaphysalis spinigera*
Alkhurma haemorrhagic fever virus	AHFV	Flaviviridae, *Flavivirus*	Positive strand RNA	Saudi Arabia	*Ornithidoros savigny*
African swine fever virus	ASFV	Asfarviridae, *Asfivirus*	Double-stranded DNA	Africa, Central Europe, Sardinia	*Ornithodoros moubata*^c^

a *In China*.

b *In North America*.

c *In Africa*.

## Emerging tick—borne bunyaviruses

### Emergence of severe fever with thrombocytopenia syndrome virus in china

Severe fever with thrombocytopenia syndrome (SFTS) was first documented in humans in 2007 when three patients were admitted to a hospital in Henan Province, China, with acute fever and severe leucopenia (Liu et al., 2014). Further cases were reported in Henan and Hubei provinces between 2008 and 2010 with a similar clinical presentation and a case-fatality rate of over 10%. In 2009, the etiological agent of these infections was isolated from a patient's blood during the outbreaks of SFTS in Xinyang City in Henan province and identified as a bunyavirus related to viruses in the genus *Phlebovirus* (Zhang et al., 2012a,b; Lam et al., 2013). The virus was subsequently named severe fever with thrombocytopenia syndrome virus (SFTSV). Phleboviruses belong to the family Bunyaviridae, characterized by viruses with a segmented single-stranded negative sense genome divided into three segments, a small (S), medium (M), and large (L) segment coding for the nucleoprotein (N), surface glycoproteins (Gn/Gc), and RNA-dependent RNA polymerase (L), respectively. Related viruses include the mosquito-borne Rift Valley fever virus (Mansfield et al., 2015), the sandfly-borne Toscana virus (Charrel et al., 2012), and the tick-borne Uukuniemi virus (Matsuno et al., 2015). At present, infections with SFTSV have been reported in at least 13 provinces in China, including Henan, Hubei, Anhui, Shandong, Jiangsu, and Zhejiang (Li, 2013). Preliminary investigation of the first outbreak revealed that patients with SFTS were predominantly individuals who worked outdoors such as farmers indicating a rural source for the infection. Numerous patients presented with a history of tick bites (Zhang et al., 2012a). Subsequently, cases of SFTS were reported in North Korea, South Korea, and Japan (Liu et al., 2014).

In order to identify the reservoir of SFTSV, ticks were collected from livestock around the localities where patients originated and SFTSV was detected in *Haemaphysalis longicornis* (Zhang and Xu, 2016). Further surveys of *H. longicornis* provided strong evidence that this tick species was the definitive reservoir and vector responsible for SFTSV transmission to humans working in rural locations (Zhang et al., 2012b). Experimental studies also demonstrated transstadial transmission of the virus between the different life stages of *H. longicornis* and transmission to mice during feeding (Luo et al., 2015). With *H. longicornis* being the source of infection and associated with feeding on livestock, it was highly likely that domestic animals would be exposed to infection and seroprevalence studies in China have now confirmed this (Zhang and Xu, 2016). However, disease in livestock has not been reported suggesting that humans are particularly susceptible to infection with SFTSV.

### Emergence of heartland virus in the USA

Heartland virus (HRTV) was first reported in 2009 from North America when two farmers from Missouri with a clinical presentation similar to SFTS were admitted to hospital (McMullan et al., 2012). Symptoms included fever, fatigue, headache, myalgia, arthralgia, anorexia and diarrhea. The source of the infection was identified as likely resulting from tick bites, and the isolated virus shared 73% sequence identity with SFTSV by alignment of the polymerase gene. Diagnosis of HRTV infection may be challenging but can be achieved through detection of viral RNA in blood or tissue or by demonstrating a four-fold or greater increase in virus-specific plaque reduction neutralization antibody titre between acute and convalescent serum specimens (McMullan et al., 2012). Seroprevalence studies of livestock in Minnesota State reported seropositivity rates ranging from 10 to 18%, for example 15.5% in cattle, suggesting widespread exposure to infected ticks (Xing et al., 2013).

Surveillance for HRTV in Missouri has identified virus in the lone star tick *Amblyomma americanum* (Savage et al., 2016), an abundant tick species in many regions of the United States. Transmission of virus to a vertebrate host, transstadial and transovarial transmission between lifecycle stages has been demonstrated in *A. americanum* (Godsey et al., 2016). Both SFTSV and HRTV are related phleboviruses and have emerged in the past 10 years. However, the viruses exist in different tick species and have emerged in different continents. Human encroachment, probably combined with environmental conditions that influence both tick abundance and behavior may have led to the simultaneous emergence of these viruses.

### The spread of crimean congo haemorrhagic fever virus around the Mediterranean Basin

The virus now known as Crimean Congo Haemorrhagic fever virus (CCHFV) was first described during an outbreak of haemorrhagic fever in Red Army soldiers in the Crimea during the final years of the Second World War (Ergönül, 2006). This virus was shown to be antigenically similar to a second virus isolated from a human in what is now the Democratic Republic of the Congo (Simpson et al., 1967). Worldwide, many tick species have been found infected with CCHFV and could be implicated in transmission of this virus (Mertens et al., 2013). The virus is primarily transmitted by ticks of the genus *Hyalomma* (Morikawa et al., 2007). By the late twentieth century, CCHFV had one of the most extensive distributions of any tick-borne virus with infections being reported from the Middle East, Asia, and sub-Saharan Africa. However, during the late twentieth and early twenty-first century, cases of CCHFV have been reported in new geographical locations around the Mediterranean Basin. CCHFV belongs to the family Bunyaviridae, genus *Nairovirus*. Other members of this genus include Hazara virus and Nairobi sheep disease virus. The virus genome is a tri-segmented negative sense RNA genome that encodes four structural proteins, the nucleoprotein (N), glycoproteins (GN and GC), and the RNA-dependent RNA polymerase (L). The virus is particularly virulent for humans but has little or no pathogenic effect on livestock. Infection following a tick bite is followed by a short incubation period of between 3 and 7 days leading to a brief febrile illness characterized by fever, headache and myalgia. Haemorrhagic manifestations of disease develop 4–5 days after the development of fever and include bleeding from the nose, gastrointestinal system and urinary tract. The fatality rate ranges from 3 to 30%. Treatment is supportive, including replacement of fluids and blood constituents. There is no licensed antiviral treatment or licensed vaccine for CCHF.

Reports of infection around the Mediterranean Basin began around 2002 with cases reported in Turkey (Karti et al., 2004) and the Balkans (Papa et al., 2005). In Europe, the main tick vector is *Hy. marginatum*, a species found throughout the Iberian Peninsula, southern France, Italy, the Balkans, and Turkey. In 2010, the virus was detected in *Hy. lusitanicum* ticks in Spain (Estrada-Peña et al., 2012), followed 6 years later with two human cases of autochthonous transmission (Garcia Rada, 2016). Phylogenetic analysis of virus detected in ticks indicated that the virus present in Spain shared greater identity with CCHFV in West Africa than that spreading in the Balkans. This suggested that it was a separate introduction into Europe with the most likely mechanism of entry being by tick-infested migrating birds (Estrada-Peña et al., 2012; Palomar et al., 2016). *Hy. marginatum* have been detected on birds migrating into Europe on numerous occasions (Capek et al., 2014) including as far north as the United Kingdom (Jameson et al., 2012). It is likely that the severity of the winters prevents establishment of this tick species in countries of northern Europe. However, the existence of populations of *Hyalomma* spp. in Spain, Portugal, France, and Italy puts these countries at greater risk of CCHFV persisting if introduced.

## Emerging tick-borne flaviviruses

### Interstate spread of Kyasanur Forest virus in India

Kyasanur Forest disease virus (KFDV) was isolated following an outbreak of haemorrhagic fever amongst villagers living in the Kyasanur Forest area in 1957 (Work and Trapido, 1957). The Forest is found in the Shimoga distict of Karnataka State in the south-west of India and has been the epicenter of the disease ever since with between 400 and 500 human cases reported annually (Holbrook, 2012). Initial studies demonstrated that the virus shared similar properties to Russian spring summer encephalitis virus (RRSEV), a variant of tick-borne encephalitis virus. However, the main clinical manifestation of disease is haemorrhagic fever unlike the encephalitis associated with RSSEV. KFDV is a member of the genus *Flavivirus*, family Flaviviridae. The flaviruses consist of a positive-sense single-stranded RNA genome that codes for three structural and seven non-structural proteins. Virion particles are enveloped with a single envelope protein projecting from the virus. KFDV is closely related to Alkhurma virus (AHFV) (Charrel et al., 2007) and a virus isolated in Yunnan Province, China (Wang et al., 2009). Phylogenetic studies on KFDV and AHFV suggest that they diverged over 700 years ago (Dodd et al., 2011) and that the virus has spread slowly within Karnataka Forest, reflecting dissemination by the tick vector that primarily feeds on small to medium sized mammals. Long distance movement that has separated the progenitors of both viruses could have been mediated by infected ticks attaching to migrating birds (Mehla et al., 2009).

The association with tick-borne viruses suggested a tick vector as the source of infection. Another early observation was mortality of non-human primates such as macaques and langurs in the area where human cases were reported. *Haemaphysalis* spp. ticks were collected from primate carcases and KFDV was isolated from *Haemaphysalis spinigera* (Trapido et al., 1959). Following the detection of KFDV, a second virus, Kaisodi virus, was isolated from *H. spinigera* sampled in India (Bhatt et al., 1966; Pavri and Casals, 1966). *Haemaphysalis* spp. are three-host ticks with a larval, nymphal and adult stage, each taking a blood meal prior to metamorphosis into the next stage, or egg development in the case of adult females. Although transovarial transmission of KFDV has been demonstrated in *H. spinigera* (Singh et al., 1963), it is the nymph and adult stages that are critical for the transmission of virus to small mammals and humans as these are the hosts favored by these life stages of the tick.

In recent years there have been reports of KFDV infections in both Karnataka province (Mourya et al., 2013; Yadav et al., 2014) and in the neighboring provinces of Kerala (Tandale et al., 2015; Sadanandane et al., 2017), Tamil Nadu and Maharashtra (Mourya and Yadav, 2016). As in previous reports, outbreaks are often preceded by disease in monkeys, and contact with carcases can lead to infection (Mourya et al., 2013). The appearance of dead monkeys, particularly target species such as the red-face bonnet monkey (*Macaca radiata*) and the black-faced langur (*Semnopithecus entellus*) are considered sentinels for the presence of KFDV at a site (Murhekar et al., 2015). The spread of KFDV in recent years could reflect further gradual spread of the virus by the tick vector, improvements in diagnosis (Mourya et al., 2012) and surveillance leading to increased frequency of reporting. Increased human exploitation of the environment leading to greater contact between humans and ticks could also contribute to this apparent spread. All of the affected regions form part of the Western Ghats, a mountain range running north-south parallel to the west coast of India. This in turn suggests that the areas affected share climatic features that are favorable to the tick vector, currently poorly studied, and that its presence could indicate a risk of KFDV. Further surveillance assessing the distribution and abundance of *H. spinigera* could reveal the true extent of KFDV in India.

### Emergence of Alkhurma haemorrhagic fever in Saudi Arabia

Alkhurma haemorrhagic fever virus (AHFV) is a recently described tick-borne virus within the genus *Flavivirus* and family Flaviviridae (Horton et al., 2016). AHFV was first isolated in 1995 from a patient with haemorrhagic manifestations and fever in the city of Alkhurma in Saudi Arabia (Zaki, 1997). The whole genome of AHFV has been derived, and shares 89% sequence identity with KFDV (Charrel et al., 2001). This suggests that AHFV is a variant genotype of KFDV but with a distinct geographical distribution. AHFV is classified as biosafety level 3 agent (Charrel et al., 2007).

To investigate the tick-borne nature of AHFV, ticks were collected in both western and southern Saudi Arabia and assessed by reverse transcriptase PCR (Eraksoy, 2015). At present, AHFV has been identified in both the *Argasid* tick *Ornithodoros savignyi* and the *Ixodid* tick *Hyalomma dromedarii* (Charrel et al., 2007). As a recently emerged virus, the geographic range of AHFV is poorly understood. The wide distribution of *Ornithodoros* and *Hyalomma* spp. ticks suggests that the geographic limits of AHFV may be larger than presently assumed. The clinical case from Najran and report of AHFV in ticks from the Horn of Africa supports this view (Memish et al., 2005; Horton et al., 2016).

### Increased human incidence of Powassan virus in New England

Powassan virus (POWV) causes fatal encephalitis in a proportion of humans that become infected with it. Between 2013 and 2015, 8 cases of POWV encephalitis were reported from hospitals in Massachusetts and New Hampshire (Piantadosi et al., 2016). This represented the most recent evidence of an increasing trend for human cases of POWV in the United States where 9 cases were reported between 1999 and 2005 (Hinten et al., 2008). Similar trends were observed for other tick-borne diseases such as Lyme borreliosis (Bacon et al., 2008) and infection with *Babesia microti* (Vannier et al., 2015). POWV is a flavivirus, causing a febrile disease that can develop into severe meningoencephalitis. The virus was first reported from a fatal case in a 5 year old child in Powassan, Ontario in 1958 (McLean and Donohue, 1959).

Preliminary virological analysis suggested an association with tick-borne viruses such as (RSSEV) pointed researchers in the direction of ticks as the vector. Subsequent field surveillance detected POWV in pools of *Ixodes cookei* ticks and provided evidence that small mammals such as groundhogs (*Marmota monax*) contribute to the maintenance of virus (McLean et al., 1967). Surveillance by groups in the United States detected POWV in Colorado (Thomas et al., 1960) and New York State (Whitney and Jamnback, 1965). Numerous examples of POWV have now been isolated from the black-legged tick *I. scapularis* (Anderson and Armstrong, 2012) indicating that this tick species may be the most abundant vector of the virus across the eastern states of the US and southern states of Canada. In a further twist, POWV has been reported from the eastern province of Russia, Primorsky Krai, and is suspected of being established across a larger geographical area (Leonova et al., 2009; Deardorff et al., 2013). Here, the tick vector includes *Ixodes* spp. such as *I. persulcatus*. Phylogenetic analysis of North American POWV strains indicates that POWV diverged from a common ancestor present ~500 years ago (Pesko et al., 2010). The viruses in Russia could be an introduction due to their similarity with American isolates. One possible means of introduction of the virus could be the importation of North American species such as mink (*Neovison vison*) with infected ticks during the expansion of the Russian fur trade.

The number of human cases of Powassan virus infection across its range has increased in recent years. The underlying cause is unclear but could be due to an increased awareness amongst clinicians and diagnosticians. Alternatively, the increase could be related to ecological factors that lead to the increase in abundance of the tick population driven in turn by increases in the number and range of mammalian hosts such as deer. This is believed to have been behind the increase in Lyme disease, caused by *B. burgdorferi*, across many States in the USA (Barbour and Fish, 1993). However, the nymphal form of the tick is suspected of being responsible for most cases of transmission and abundance of this life stage is more dependent on the availability of small mammals such as rodents, for example the white-footed mouse (*Peromyscus leucopus*). Therefore, factors that influence rodent abundance such as predator decline, mediated by the red fox (*Vulpes vulpes*) for example, may have greater impact on disease transmission by ticks (Levi et al., 2012).

### The spread of the deer tick virus in North America

Deer tick virus (DTV), a flavivirus in the tick-borne encephalitis group, is a genetically distinct lineage (subtype) of POWV that can cause neuroinvasive infection in humans in parts of North America. DTV was originally isolated from the Rocky Mountain wood tick, *Dermacentor andersoni*, but is mainly found in *I. scapularis* collected from states in the north-east of the US (Telford et al., 1997; Aliota et al., 2014) and has since been responsible for a human case of encephalitis (Tavakoli et al., 2009). Although the first recognized human case of DTV encephalitis occurred in 1997, evidence of the causative virus, based on sequence data, was not available until 2001 (Gholam et al., 1999; Kuno et al., 2001). The DTV genome shares 84% sequence identity with POWV and 94% amino acid identity between the virus polyproteins. However, DTV and POWV are regarded as antigenically indistinguishable and the infecting virus cannot be determined by serological testing, and genotypic analysis is needed to make a definitive diagnosis. DTV is maintained in an enzootic cycle between *I. scapularis* and the white-footed mouse (*Peromycus leucopus*) (Ebel et al., 2000). Until now there have been a small number of published cases of proven DTV induced encephalitis (Kuno et al., 2001), one from Canada and three from USA. Based on detection of virus genome, prevalence with DTV ranged up to 5% in *I. scapularis* from several geographic areas including Hudson Valley, Nantucket Island and Prudence Island (Aliota et al., 2014). The increase in the number of human cases of Powassan virus encephalitis that have been reported since 2010 is remarkable. Most of these cases were diagnosed by serological assays (Khoury et al., 2013) and it is possible that DTV could be responsible for some of these cases.

## The emergence of Asfarvirus in Europe

### Establishment of African swine fever virus in Eastern Europe

African swine fever virus (ASFV) is the causative agent of African swine fever (ASF), an acute haemorrhagic fever that causes severe morbidity and high mortality in domestic pigs. ASFV is classified within the genus *Asfivirus* and family Asfarviridae, and consists of a double-stranded DNA genome coding for ~150 proteins. The genome is surrounded by a protein capsid and a host-derived envelope. Virus particles are very robust and can survive for days in the environment or months within pork meat. ASF was first described in Kenya and is found across sub-Saharan Africa (Thomson, 1985). The epidemiology of ASF in Africa is driven by two cycles. The first is sylvatic with bush pigs (*Potamochoerus larvatus*) and warthogs (*Phacochoerus africanus*) being infected following infestation with *Argasid* ticks of the genus *Ornithodorus*, particularly *O. moubata*. Infections in wild species do not result in clinical signs of disease. The second cycle involves infection of domestic pigs (*Sus scrofa*) that are highly susceptible to infection and shed virus in excreta. Initial infection is by tick bite but is then amplified by pig-to-pig transmission, either through contact or consumption of contaminated food. ASF is a notifiable disease in Europe (http://www.oie.int/animal-health-in-the-world/oie-listed-diseases-2016/) and significant effort is directed at preventing its introduction through import control of livestock and food stuffs. Outbreaks resulting from importation have occurred in domestic pigs on a number of occasions across Europe. Significantly in the case of Sardinia, following introduction in the late 1970s. Repeated attempt to eliminate the disease have failed and the disease is endemic (Mur et al., 2016). More recently, ASFV was introduced into the Caucasus region in 2007 (Rowlands et al., 2008) and led to extensive spread into neighboring countries including Armenia, Azerbaijan, and Russia. By 2016, ASFV has been detected throughout Eastern Europe and the Baltic region. Pig-to-pig transmission appears to be the main driver for the spread of the epidemic but wild boar, a species which is also susceptible to infection, could also contribute to spread (Guinat et al., 2016). A further concern for Europe is the infection of indigenous *Argasid* ticks such as *O. erraticus* that are present throughout the Mediterranean Basin and Balkans, and feed preferentially on domestic pigs, although less so on wild boar (Pietschmann et al., 2016). ASFV infects and replicates in *O. erraticus* (Basto et al., 2006; Ribeiro et al., 2015) and has been shown to experimentally transmit ASFV to pigs (Boinas et al., 2011). Widely distributed *Ixodid* ticks in Europe such as *I. ricinus* and *Dermacentor reticulatus* are unable to support ASFV replication and presumably do not contribute to disease spread (de Carvalho Ferreira et al., 2014). The spread of ASFV has been primarily caused by human activities including long distance transport of livestock. The presence of a susceptible wildlife host, wild boar, has further complicated efforts to control the disease and it is likely that it will continue to spread across the continent.

## Tick saliva-assisted transmission and potential antigenic targets for control

The components of saliva are critical to the successful completion of a blood meal by all life stages of ticks. As a result, tick saliva is a highly complex mixture of proteins, peptides, and other bioactive compounds. Transcriptomic analysis suggests that tick feeding leads to the upregulation of thousands of protein transcripts (Karim and Ribiero, 2015; Ribeiro et al., 2017) that when secreted promote attachment to the host, inhibit host responses such as blood clotting and inhibit microbial growth (Hovius et al., 2008). Tick saliva has also been shown to promote transmission of tick-borne viruses to the mammalian host. Early studies demonstrated that salivary gland extracts (SGE) from a range of tick species enhanced transmission of Thogoto virus and TBEV to guinea-pigs (Jones et al., 1989, 1992; Labuda et al., 1993). Recent studies have shown a similar enhancement by *O. porcinus* SGE of ASFV infection in pigs (Bernard et al., 2015) and *I. scapularis* SGE of POWV infection in mice (Hermance and Thangamani, 2015). One mechanism suggested for this enhancement is the increased attraction of macrophages and other antigen presenting cells to the site of tick attachment. These appear to rapidly disseminate the virus throughout the mammalian host. It is also possible that rather than being passively transmitted by the tick, viruses actively modulate tick salivary gland transcripts. In a study investigating feeding by *I. scapularis* nymphs, infection with Langat virus modulated tick salivary gland transcriptional responses during 3 days of feeding (McNally et al., 2012). Further analysis of virus-induced transcripts could lead to the identification of those proteins that promote virus transmission and in turn could be molecular targets for tick control and prevention of virus transmission. The concept of a tick vaccine has been discussed for decades and has led to a number of approaches based on specific tick salivary proteins that have been used as vaccine formulations that inhibit tick feeding (Labuda et al., 2006; Garcia-Varas et al., 2010). The advent of “omics” technologies using both the sialotranscriptome (Maruyama et al., 2017) and the proteome (Villar et al., 2017) have dramatically expanded the number of targets available for vaccine development. The application of such vaccines to livestock or even key wildlife species could provide an opportunity to suppress tick abundance and reduce the frequency of pathogen transmission in order to suppress incidence of disease.

## Conclusions

The emergence of tick-borne viruses is driven by a range of factors, often inter-related, that lead to the appearance and/or increase in human or veterinary cases of disease. *Ixodid* tick species have multiple life stages (Figure 1) with each feeding off a different host, and often a different host species. Factors that influence each life stage will affect their ability to transmit pathogens. Key amongst these factors are those associated with the tick vector, including its presence in an area, feeding behavior, abundance, and contact with humans or livestock. Studies on TBEV suggest that co-feeding favors virus transmission between immature tick stages (larva/nymphs) and environmental factors that promote co-feeding will drive infection rates up via horizontal transmission and increase the risk of transmission to humans (Randolph et al., 1999). This leads to the paradoxical situation whereby decreases in numbers of mammalian hosts can lead to an increase in disease transmission. Seasonal variation in temperatures can also dramatically affect transmission to humans. Low temperatures in winter and warm summer temperatures between 2009 and 2012 have been identified as the reason for the increased incidence of TBEV in Sweden enhanced by co-feeding by *I. ricinus* on small mammals (Jaenson et al., 2012). Warm summer temperatures also encourage humans to spend more time outdoors and wear less clothing, both factors that could increase the risk of encountering ticks. The presence of large ruminants such as deer can increase the abundance of ticks and indirectly indicate risk of virus transmission (Carpi et al., 2008). Conversely, activities that reduce tick abundance will reduce virus transmission.

The distribution of a tick-borne disease is usually dictated by that of the tick vector. This appears to be the case for viruses such as CCHFV. However, when driven by anthropomorphic factors, such viruses can emerge in new locations, enabled by an alternative mechanism of transmission. This appears to have led to the emergence of ASFV in Central Europe where tick-borne transmission has been replaced by contact transmission between pigs, driven in part by the presence of wild boar (Guinat et al., 2016).

Human factors are a major influence on disease emergence. The paradigmatic example of this is the emergence of Omsk haemorrhagic fever virus in central Russia due to the importation of muskrats (*Ondatra zibethicus*) from North America in the nineteenth century (Ružek et al., 2010). The virus existed in the area within the *D. reticulatus* tick population but was rarely, if ever, encountered by humans. By introducing an exotic mammalian host that was highly susceptible to infection and regularly handled by humans, clusters of haemorrhagic disease occurred. A similar scenario appears to have resulted in the introduction of POWV in Eastern Russia and the activities of the farming industry in Eastern Europe has propelled the spread of ASFV. A further human factor that can increase detection rates of emerging tick-borne diseases is awareness by clinicians. Once a cluster of cases of disease have been reported, further cases come to light. However, a lack of disease awareness among clinicians and veterinarians can hamper the control of a disease epidemic, which could potentially be the case for ASFV where clinical signs can be similar to those observed with other diseases of swine (Guinat et al., 2016). Therefore, an increase in awareness and data sharing of new and emerging tick-borne diseases among medical and veterinary professionals is essential. Trans-national bodies such as the World Health Organization and the World Organization for Animal Health (OIE) play a key role in information sharing and setting standards for disease detection and reporting.

Tick-borne diseases continue to cause a burden to both animal and human health. As the twenty-first century progresses there will certainly be more examples of tick-borne virus spread and novel virus emergence. New viruses associated with ticks are being discovered as a result of human infection, such as Bourbon virus (Kosoy et al., 2015) or before there is any association with disease such as Tofla virus (Shimada et al., 2016; de Figueiredo et al., 2017). Early recognition of those that are pathogens will be critical to their control in addition to measures to control potential tick vectors. Understanding the interactions between these emerging viruses and the tick species that transmit them to vertebrate hosts represents both a colossal challenge and a great opportunity to identify targets for future control of tick-borne diseases. A greater understanding of tick feeding has identified both the complexity of the feeding process and its role in promoting pathogen transmission (Randolph, 2009). This understanding has also resulted in the identification of protein components within tick saliva that can act as antigens for vaccines that suppress tick infestation and protect against tick-borne virus transmission (Labuda et al., 2006). Such generic approaches may assist in protecting humans and livestock from emerging tick-borne viruses.

## Author contributions

NJ conceived the review. KM, LJ, LP, and NJ wrote the paper. All authors reviewed the final version of the manuscript.

### Conflict of interest statement

The authors declare that the research was conducted in the absence of any commercial or financial relationships that could be construed as a potential conflict of interest.
